# Developmental Competence of Vitrified-Warmed Bovine Oocytes at the Germinal-Vesicle Stage is Improved by Cyclic Adenosine Monophosphate Modulators during In Vitro Maturation

**DOI:** 10.1371/journal.pone.0126801

**Published:** 2015-05-12

**Authors:** Kenji Ezoe, Akiko Yabuuchi, Tetsuya Tani, Chiemi Mori, Tetsuya Miki, Yuko Takayama, Zeki Beyhan, Yoko Kato, Takashi Okuno, Tamotsu Kobayashi, Keiichi Kato

**Affiliations:** 1 Kato Ladies Clinic, Shinjuku-ku, Tokyo, Japan; 2 Laboratory of Animal Reproduction, College of Agriculture, Kinki University, Nara, Japan; 3 Sher Institute for Reproductive Medicine-Las Vegas, Las Vegas, NV, United States of America; Institute of Zoology, Chinese Academy of Sciences, CHINA

## Abstract

Cryopreservation of mature oocytes and embryos has provided numerous benefits in reproductive medicine. Although successful cryopreservation of germinal-vesicle stage (GV) oocytes holds promise for further advances in reproductive biology and clinical embryology fields, reports regarding cryopreservation of immature oocytes are limited. Oocyte survival and maturation rates have improved since vitrification is being performed at the GV stage, but the subsequent developmental competence of GV oocytes is still low. The purpose of this study was to evaluate the effects of supplementation of the maturation medium with cyclic adenosine monophosphate (cAMP) modulators on the developmental competence of vitrified-warmed GV bovine oocytes. GV oocytes were vitrified-warmed and cultured to allow for oocyte maturation, and then parthenogenetically activated or fertilized in vitro. Our results indicate that addition of a cAMP modulator forskolin (FSK) or 3-isobutyl-1-methylxanthine (IBMX) to the maturation medium significantly improved the developmental competence of vitrified-warmed GV oocytes. We also demonstrated that vitrification of GV oocytes led to a decline in cAMP levels and maturation-promoting factor (MPF) activity in the oocytes during the initial and final phases of maturation, respectively. Nevertheless, the addition of FSK or IBMX to the maturation medium significantly elevated cAMP levels and MPF activity during IVM. Taken together, our results suggest that the cryopreservation-associated meiotic and developmental abnormalities observed in GV oocytes may be ameliorated by an artificial increase in cAMP levels during maturation culture after warming.

## Introduction

The developmental competence of oocytes has been improved by modulation of cyclic adenosine monophosphate (cAMP) levels during in vitro maturation (IVM) [1]. Follicle stimulating hormone and luteinizing hormone activate G protein-coupled receptors that stimulate the production of cAMP by adenylate cyclase. cAMP acts as an intracellular messenger for gonadotropin stimulation and plays a critical role in maintaining the meiotic arrest of mammalian oocytes and in inducing their maturation [2–4]. Relatively high levels of cAMP within the oocyte are essential for maintaining the meiotic arrest, whereas a drop in the intraoocyte concentration of cAMP causes resumption of meiosis and maturation [5]. Maintenance of an appropriate cAMP concentration in oocytes is an important requirement for chromatin transition and for synchronization of nuclear and cytoplasmic maturation processes during the final oocyte maturation [1,6,7]. Some studies have shown that artificial regulation of meiotic resumption by cAMP-upregulating agents improves subsequent oocyte developmental competence in domestic animals, mice, and humans [7–11]. Additionally, a recent study showed that modulation of cAMP content during the first 1–2 h after oocyte collection is critical for oocyte development, and that this regulation can be achieved by treatment with an adenylate cyclase activator or a nonspecific phosphodiesterase inhibitor, e.g., forskolin (FSK) or 3-isobutyl-1-methylxanthine (IBMX). After this treatment, cAMP levels increase and a loss of gap junctions and resumption of meiosis are prevented synergistically, resulting in increased developmental competence [12–14].

The development of cryopreservation techniques for mature metaphase II stage (MII) oocytes has provided many benefits for fertility preservation. These techniques can be applied not only to the breeding of livestock animals but also to the clinical practice of reproductive medicine [15–17], especially for young women receiving cancer treatment. In human assisted reproductive technology (ART), for instance, it has been proven that the developmental potential of MII oocytes cryopreserved by a vitrification system are comparable to non-vitrified oocytes; therefore, these techniques are no longer considered experimental [18]. However, cryopreservation of mature oocytes poses certain technical and clinical complexities, such as the requirement for lengthy hormonal stimulation protocols for oocyte retrieval. Because many oocyte retrieval procedures depend on the patient’s menstrual cycle, setting the appropriate timing of oocyte retrieval prior to cancer treatment may be challenging in cancer patients. By contrast, recovery of germinal-vesicle stage (GV) oocytes followed by IVM is a potentially useful procedure for the generation of mature oocytes. Many GV oocytes could be recovered without exogenous gonadotropin treatment regardless of the patient’s estrus cycle, thus reducing the risk of ovarian hyperstimulation syndrome and the cost and complexity of treatment [19]. In addition, GV oocytes are theoretically more resistant to the physical damage than MII oocytes are and carry no risk of polyploidy and aneuploidies because the chromatin is diffuse in the diplotene state of prophase I and is surrounded by a nuclear membrane, which may prevent spindle depolymerization [20–22]. Therefore, it is believed that GV oocytes are structurally more suitable for cryopreservation than MII oocytes, and cryopreservation of GV oocytes has been proposed as an effective method for preservation of rare species, embryo production for livestock artificial breeding programs, the treatment of human infertility, and research on reproductive and developmental biology. Despite the clear advantages of cryopreservation of GV oocytes, difficulties still exist with embryonic development after cryopreservation. Although the rates of oocyte survival and maturation have improved in humans, bovine, and rodents, poor embryonic development is the main problem associated with cryopreservation of GV oocytes [23–26]. With the improvement of vitrification techniques, the survival rate after vitrification at the GV stage was found to be comparable to that at the MII stage. However, the embryo developmental competence is significantly reduced by vitrification at the GV stage; therefore, it has been recommended that oocytes should be vitrified at the mature MII stage after IVM rather than at the immature GV stage [27–30]. Therefore, the improvement of the developmental competence of oocytes vitrified-warmed at the GV stage could significantly contribute to successful implantation during livestock breeding and to endangered-species preservation programs in addition to human assisted-reproduction technologies.

In this study, to improve the developmental competence of oocytes vitrified-warmed at the GV stage, we addressed whether maintenance of the cAMP concentration in oocytes during IVM by cAMP modulators improves the poor embryonic development caused by vitrification at the GV stage.

## Materials and Methods

### Oocyte collection

Bovine ovaries were transported from Yokohama-City Central Slaughterhouse Co. LTD. (Kanagawa, Japan) to the laboratory in saline at 12°C within 24 h after slaughter of the animals. These bovine were slaughtered for the meat product not for the experiments. Therefore, the ethical approval from our institutional ethical committee was not needed. GV oocytes surrounded by cumulus cells were aspirated from follicles (2–8 mm diameter) using an 18-gauge needle attached to a 10-mL syringe at room temperature. The GV oocytes with three or more layers of cumulus cells and a homogeneous cytoplasm were selected for the experiment. These were washed three times in the HEPES-buffered tissue culture medium 199 (TCM-199; Sigma-Aldrich, St. Louis, MO, USA) supplemented with 1% serum substitute supplement (SSS, Irvine Scientific, Santa Ana, CA, USA), abbreviated here as mTCM-199/1% SSS. Approximately 60–70% of cumulus cells were removed from the oocytes by pipetting and were immediately subjected to vitrification/warming or an IVM process.

### Vitrification and warming

The GV oocytes were cryopreserved by vitrification using Cryotop (Kitazato BioPharma, Shizuoka, Japan). The medium used for handling oocytes during vitrification and warming was HEPES-buffered TCM-199 supplemented with 20% SSS, abbreviated here as mTCM-199/20% SSS. All manipulations were performed at room temperature, and all the media were at room temperature, except for the warming solution, which was heated to 37°C. GV oocytes were transferred into the equilibration solution composed of 7.5% ethylene glycol (EG; Sigma-Aldrich) and 7.5% dimethyl sulfoxide (DMSO; Sigma-Aldrich) in mTCM-199/20% SSS and kept there for 15 min. This was followed by transfer to the vitrification solution composed of 20% EG, 20% DMSO, and 0.5M sucrose in mTCM-199/20% SSS for 1-min incubation at room temperature. The GV oocytes that were placed on Cryotop were submerged directly into liquid nitrogen. For warming, Cryotop was placed directly in the warming solution (composed of 1.0M sucrose in mTCM-199/20% SSS) and incubated for 1 min at 37°C. The GV oocytes detached from the Cryotop were transferred into a dilution solution (0.5M sucrose in mTCM-199/20% SSS) and incubated there for 3 min. After a wash, survival rate of the oocytes was evaluated by identifying the morphologic appearance. The surviving oocytes were used further for the subsequent experiments.

### IVM

The GV oocytes were cultured for 22 h in IVMD (Research Institute for the Functional Peptides, Yamagata, Japan). Depending on individual experimental design, GV oocytes were cultured in IVMD supplemented with the forskolin (FSK, 100 μM; Sigma-Aldrich), 3-isobutyl-1-methylxanthine (IBMX, 100 μM; Sigma-Aldrich) or the combination of FSK and IBMX for 22, 26, or 30 h according to a previous report, with slight modifications [13,31,32]. GV oocytes were cultured at 38.5°C in a humidified atmosphere of 5% CO_2_ and 95% air, and the oocyte maturation rate was calculated judging by the first polar body extrusion in the oocytes after removal of the cumulus cells.

### Parthenogenetic activation

Mature oocytes were chemically activated in mTCM-199/1% SSS with 5 μM calcium ionophore (Sigma-Aldrich) for 5 min, rinsed three times in mTCM-199/1% SSS, and immediately incubated in the synthetic oviductal fluid medium (SOF) containing 2 mM 6-dimethylaminopurine (Sigma-Aldrich), for 6 h at 38.5°C in a humidified atmosphere consisting of 5% CO_2_, 5% O_2_, and 90% N_2_. Then, the oocytes were rinsed three times in mTCM-199/1% SSS and transferred to SOF under mineral oil (day 0). The dishes were incubated at 38.5°C in a humidified atmosphere consisting of 5% CO_2_, 5% O_2_, and 90% N_2_. Cleavage rate was recorded on day 2, and the number of embryos developing to the blastocyst stage was assessed on day 8. The experiments were repeated at least three times.

### In vitro fertilization (IVF)

Fresh and vitrified-warmed oocytes were washed three times in the fertilization medium (IVF100, Research Institute for the Functional Peptides), and the group of 10 oocytes was transferred to a 100-μL drop of IVF100. Spermatozoa were obtained by centrifugation of frozen-thawed semen with IVF100 at 200 × *g* for 5 min at room temperature. The spermatozoa were counted and diluted in the appropriate volume of the medium for fertilization. The sperm suspension was added to each fertilization drop to obtain a final concentration of 5 × 10^6^/mL. The oocytes were incubated with sperm for 6 h at 38.5°C in a humidified atmosphere of 5% CO_2_ and 95% air, and the fertilization rate was determined judging by the second polar body extrusion in the oocytes after removal of the cumulus cells. Then, the fertilized oocytes were transferred to SOF under mineral oil (day 0) and were incubated at 38.5°C in a humidified atmosphere consisting of 5% CO_2_, 5% O_2_, and 90% N_2_. The cleavage rate was recorded on day 2, and the number of embryos developing to the blastocyst stage was assessed on day 8. The experiments were repeated at least three times.

### Differential staining

The quality of blastocysts was assessed according to the cell number by performing differential staining of the inner cell mass (ICM) and trophectoderm (TE) as described in a previous study, with slight modifications [33]. Briefly, blastocysts were first incubated in mTCM199/1% SSS with 0.1% (v/v) Triton X-100 (Sigma-Aldrich) and 10 μg/mL propidium iodide (Sigma-Aldrich) for 30 seconds. After rinsing in mTCM199/1% SSS, blastocysts were stained with 1 μg/mL Hoechst 33342 supplemented mTCM199/1% SSS, and then the stained blastocysts were mounted on glass slides under a cover slip and examined under a fluorescence microscope. The ICM nuclei labeled with Hoechst appeared blue, and the TE cell nuclei labeled with Hoechst and propidium iodide appeared pink.

### Examination of nuclear status of GV oocytes

The cumulus cells were removed from oocytes by gentle pipetting at 0, 0.5, 1, 2, 4, 8, 12, 22, 26, and 30 h after the initiation of the IVM culture period, and the status of germinal vesicle breakdown (GVBD) and oocyte maturation was assessed by staining in mTCM-199/1% SSS supplemented with 1 μg/mL Hoechst 33342 (Sigma-Aldrich) with subsequent fluorescence microscopy.

### ELISA

cAMP content and maturation promoting factor (MPF) activity of fresh and vitrified-warmed GV oocytes during IVM culture were measured by ELISA.

For the measurement of cAMP content, the oocytes were collected from the culture medium at 0, 0.5, 1, 2, 4, 6, and 8 h; for the measurement of MPF activity, the oocytes were collected from the culture medium at 0, 4, 8, 12, 22, 26, and 30 h after the initiation of IVM culture. Furthermore, to investigate whether the duration taken for vitrification/warming procedure (0.5 h) would affect the cAMP content of vitrified-warmed GV oocytes, the oocytes were randomly allocated to seven groups: Group 1, the oocytes collected at the initiation of IVM culture (0 h); Group 2, the oocytes cultured in mTCM-199/20% SSS at room temperature for 0.5 h; Group 3, the oocytes immersed into the vitrification/warming solution; Group 4, the oocytes vitrified and warmed using the vitrification/warming solution without FSK and IBMX; and Group 5–7, the oocytes vitrified and warmed using the vitrification/warming solution supplemented with FSK, IBMX, or the combination of FSK and IBMX, respectively.

Cumulus cells around the oocytes were completely removed, and then 10 oocytes from each group were transferred to a 0.2-mL tube with lysis buffer and incubated for 1 min. Next, they were centrifuged (500 × *g*) for 5 min and the supernatant was collected. Intra-oocyte cAMP level and MPF activity were measured using the Cyclic AMP EIA Kit (Cayman, Ann Arbor, MI, USA; for cAMP) and CycLex Cdc2-cyclin B Kinase Assay Kit (CY‑1164; CycLex, Nagano, Japan; for MPF), respectively, according to a previous report, with slight modifications [34–36]. The plates were analyzed on an iMark microplate absorbance reader (420nm Bio-Rad, Tokyo, Japan). The experiments were repeated three to five times.

### Statistical analysis

Data on the survival rate, maturation rate, fertilization rate, cleavage rate, blastocyst formation rate, cell number, cAMP concentration, and MPF activity were normally distributed and were analyzed using one-way analysis of variance (ANOVA). The significance of differences was assessed using the Fisher protected least significant difference *post hoc* multiple comparison test. The chi-squared test was used to evaluate differences in the proportion of oocytes at the GVBD stage and at the MII stage. Comparison with expected values of less than 5 was analyzed using the Fisher exact probability test. *P* < 0.05 was assumed to indicate statistical significance

## Results

### FSK and IBMX significantly improved the developmental competence of vitrified-warmed GV oocytes after parthenogenetic activation

Mature oocytes generated from vitrified-warmed GV oocytes were artificially activated to assess their developmental competence (Table 1). The maturation rate of control fresh (non-vitrified) GV oocytes was comparable with fresh FSK and IBMX groups. When the GV oocytes were vitrified and warmed, the maturation rate was significantly lower than those in fresh GV oocytes, and these values were not recovered upon the addition of FSK or IBMX. Similarly, the blastocyst formation rate per initial GV oocytes in the vitrified-warmed control group was significantly lower than that of the fresh control group. These rates were notably increased in the vitrified-warmed groups when the GV oocytes were treated with FSK or IBMX after vitrification/warming. In the FSK/IBMX-supplemented groups, low maturation rates were observed and the blastocyst formation rate was significantly lower than that in fresh control group.

**Table 1:** The survival rate and embryo development of vitrified-warmed germinal vesicle stage (GV) oocytes after parthenogenetic activation.

Experimental Groups	GV	Oocytes	Oocytes	Embryos	Blastocysts	Cell number (Mean±SEM)		
	oocytes	survived	matured	cleaved		Total	Trophectoderm	Inner cell mass
	n	n, (%)	n, (%)	n, (%)	n, (%)	(n)		
Control-Fresh	315	315	266	240	126	122.2±2.8 ^a^	90.9±2.1 ^a^	31.3±0.8 ^a^
		(100)^a^	(83.5±1.5) ^a^	(74.9±1.6) ^a^	(38.7±2.7) ^a,b^	(n = 64)		
Control-Vitrification	315	295	228	157	39	103.8±2.8 ^b^	77.8±2.1 ^b^	26.0±1.0 ^b^
		(90.3±1.5) ^b^	(70.0±2.2) ^b^	(47.7±3.2) ^b^	(10.8±1.1) ^c^	(n = 39)		
FSK-Fresh	315	315	270	241	136	123.8±2.7 ^a^	91.5±2.2 ^a^	32.3±0.8 ^a^
		(100)^a^	(84.7±2.1) ^a^	(74.2±2.8) ^a^	(40.2±3.1) ^a^	(n = 67)		
FSK-Vitrification	315	300	240	208	100	121.4±2.4 ^a^	90.5±2.1 ^a^	30.9±0.9 ^a^
		(91.9±1.6) ^b^	(74.2±2.5) ^b^	(63.3±3.8) ^c^	(31.7±3.3) ^b^	(n = 62)		
IBMX-Fresh	320	320	274	250	130	122.0±2.9 ^a^	90.8±2.2 ^a^	31.2±0.9 ^a^
		(100)^a^	(85.8±1.5) ^a^	(78.8±2.0) ^a^	(42.1±1.7) ^a^	(n = 64)		
IBMX-Vitrification	320	295	243	222	100	122.0±2.4 ^a^	91.1±1.9 ^a^	30.9±1.0 ^a^
		(92.5±1.1) ^b^	(74.3±3.9) ^b^	(66.9±4.4) ^a,c^	(33.2±2.1) ^a,b^	(n = 59)		
FSK/IBMX-Fresh	90	90	44	26	10	120.3±8.7 ^a^	89.0±6.2 ^a^	31.3±2.7 ^a^
		(100) ^a^	(50.8±2.8) ^c^	(30.8±2.8) ^d^	(10.8±0.8) ^c^	(n = 10)		
FSK/IBMX-Vitrification	90	82	37	22	8	123.8±9.5 ^a^	92.4±7.6 ^a^	31.4±2.4 ^a^
		(90.8±0.8) ^b^	(41.7±1.7) ^c^	(26.7±3.7) ^d^	(8.3±1.7) ^c^	(n = 8)		

Values within each column with different superscripts (a, b, c, d) are significantly different from each other (*P* < 0.05).

The total cell number of blastocysts and the number of TE cells and ICM cells in the vitrified-warmed control group were significantly lower than those of the fresh control group (Table 1). Nonetheless, the cell number of blastocysts derived from the vitrified-warmed FSK and IBMX groups was comparable with that of all fresh groups. These results indicated that vitrification and warming of GV oocytes negatively affected oocyte maturation and developmental competence, but these aspects were improved with the supplementation of FSK or IBMX. Moreover, the supplementation of the combination of FSK and IBMX did not improve the developmental competence with low maturation rate.

### FSK and IBMX tended to increase the developmental competence of vitrified-warmed GV oocytes after IVF

The fertilization, cleavage, and blastocyst formation rates in the vitrified-warmed control group were significantly lower than those in the fresh control group (Table 2). Although, no significant differences were observed among the vitrified-warmed groups, the number of blastocysts developed in the vitrified-warmed GV oocytes treated with FSK or IBMX tended to increase when compared to vitrified-warmed control GV oocytes, and these rates were comparable to those of the fresh groups. The total cell number of blastocysts and the number of TE cells and ICM cells were similar among all experimental groups (Table 2). These results indicated that the addition of FSK or IBMX to the IVM medium tended to improve the developmental competence of vitrified-warmed GV oocytes after IVF.

**Table 2:** Fertilization and embryo development of vitrified-warmed germinal vesicle stage (GV) oocytes after in vitro fertilization.

Experimental Groups	GV	Oocytes	Oocytes	Embryos	Blastocysts	Cell number (Mean±SEM)		
	oocytes	survived	fertilized	cleaved		Total	Trophectoderm	Inner cell mass
	n	n, (%)	n, (%)	n, (%)	n, (%)	(n)		
Control-Fresh	191	191	158	118	55	122.0±6.9	91.1±5.5	30.9±2.4
		(100) ^a^	(84.4±2.1) ^a^	(65.0±3.4) ^a^	(27.9±2.4) ^a^	(n = 16)		
Control-Vitrification	214	199	118	56	13	106.8±10.8	81.0±8.9	25.8±2.5
		(90.9±2.0) ^b^	(55.9±4.8) ^b^	(27.0±3.7) ^b^	(6.2±2.2) ^b^	(n = 6)		
FSK-Fresh	177	177	139	103	53	122.5±5.5	92.5±4.7	30.1±1.6
		(100) ^a^	(78.2±2.6) ^a^	(59.9±2.8) ^a^	(29.9±6.9) ^a^	(n = 20)		
FSK-Vitrification	216	200	128	86	39	118.8±7.9	88.7±6.1	30.1±2.3
		(92.3±0.9) ^b^	(60.4±5.4) ^b^	(40.3±4.7) ^c^	(18.6±5.9) ^a,b^	(n = 16)		
IBMX-Fresh	176	176	133	105	55	118.6±6.3	89.0±4.6	29.5±2.1
		(100) ^a^	(75.0±2.2) ^a^	(59.7±1.7) ^a^	(30.2±6.3) ^a^	(n = 22)		
IBMX-Vitrification	216	197	121	79	36	120.2±7.7	90.2±6.4	30.0±2.0
		(90.6±1.2) ^b^	(56.2±3.6) ^b^	(36.5±4.9) ^b,c^	(17.9±6.5) ^a,b^	(n = 18)		
FSK/IBMX-Fresh	50	50	17	14	9	123.9±10.6	92.3±7.5	31.6±3.3
		(100) ^a^	(35.8±9.2) ^c^	(30.0±1.0) ^b,c^	(18.3±1.7) ^a,b^	(n = 9)		
FSK/IBMX-Vitrification	50	45	11	9	7	111.6±10.5	84.0±8.6	27.6±3.0
		(90.0±0.2) ^b^	(23.3±6.7) ^c^	(19.2±5.8) ^d^	(14.2±0.8) ^b^	(n = 7)		

Values with different superscripts (a, b, c, d) within each column are significantly different from each other (*P* < 0.05).

### Treatment with FSK or IBMX induced a delay of meiotic maturation in vitrified-warmed GV oocytes

**Fig 1:**
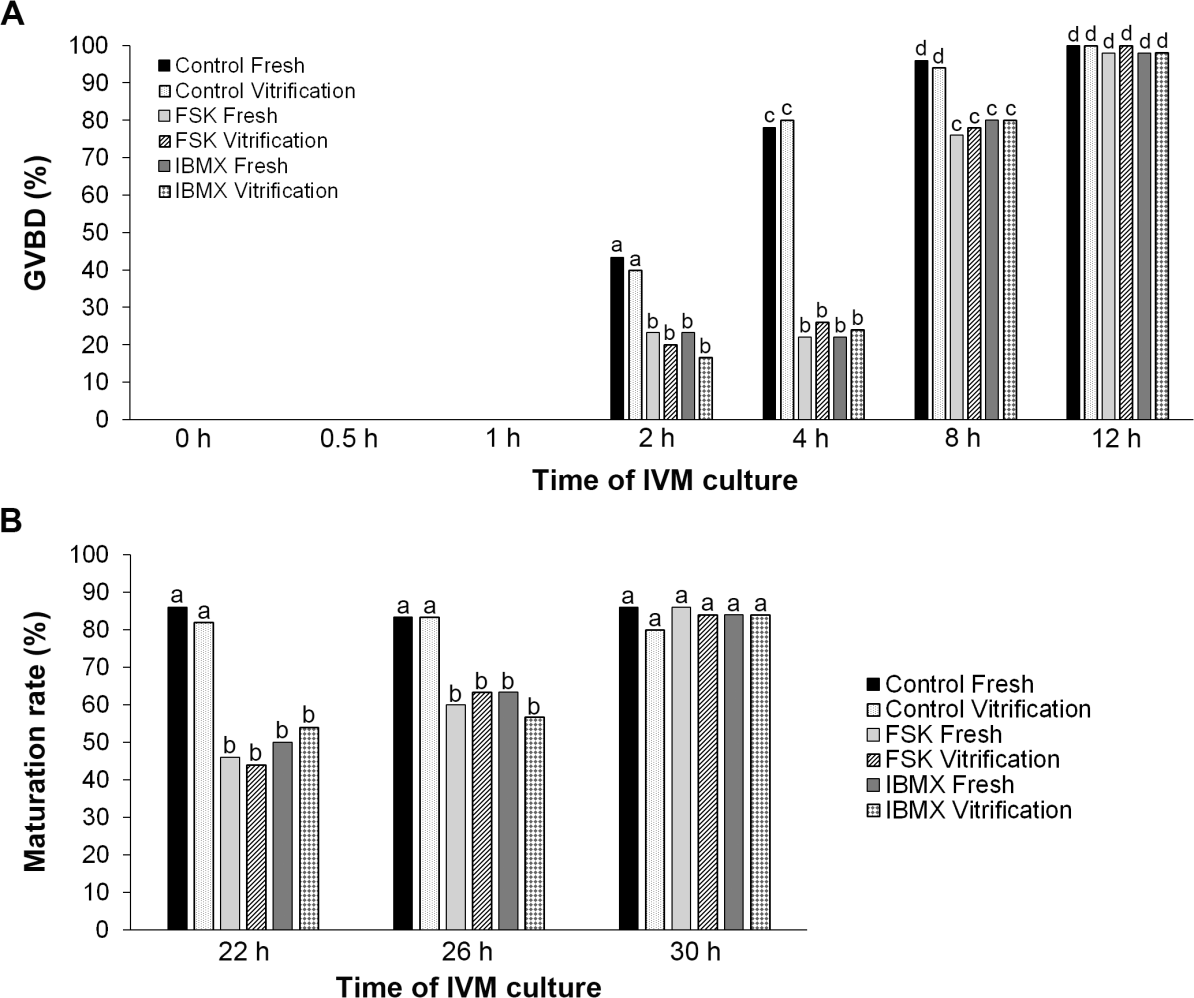
Delayed initiation of germinal vesicle breakdown (GVBD) and meiotic maturation in the oocytes treated with forskolin (FSK) and 3-isobutyl-1-methylxanthine (IBMX). *A)* The GVBD rate at 0, 0.5, 1, 2, 4, 8, and 12 h in IVM culture. Delayed GVBD was observed in the FSK and IBMX groups. *B)* The maturation rate at 22, 26, and 30 h after the initiation of IVM culture. Although delay in oocyte maturation occurred in the FSK and IBMX groups, the maturation rates at 30 h were comparable to those in the control groups. Error bars represent standard error of the mean. Bars labeled with different letters show significant differences (*P* < 0.05).

The GVBD of the fresh and vitrified-warmed control group started 2 h after the initiation of IVM culture and plateaued after 8 h of culture (Fig 1A). In the FSK and IBMX groups, although the GVBD of both fresh and vitrified-warmed oocytes was observed 2 h after initiation of IVM culture, the rate plateaued at 12 h, indicating that supplementation of the IVM medium with FSK and IBMX notably delayed GVBD in fresh and vitrified-warmed GV oocytes.

The rate of maturation to the MII stage at 22 h in the vitrified-warmed control group was comparable to that of the fresh control group (Fig 1B). The MII rates in the FSK and IBMX groups were significantly lower than that in the control group at 22 and 26 h, but these rates increased up to a rate similar to that in the control group at 30 h after initiation of IVM. These results indicated that the maturation rate was not affected by vitrification at the GV stage. Moreover, supplementation of FSK or IBMX in the IVM medium with delayed the onset of maturation by 8 h, but the maturation rate at the end of IVM (30 h) was comparable to that of the control group.

### Delayed initiation of cAMP waves in vitrified-warmed GV oocytes upon FSK or IBMX treatment

**Fig 2:**
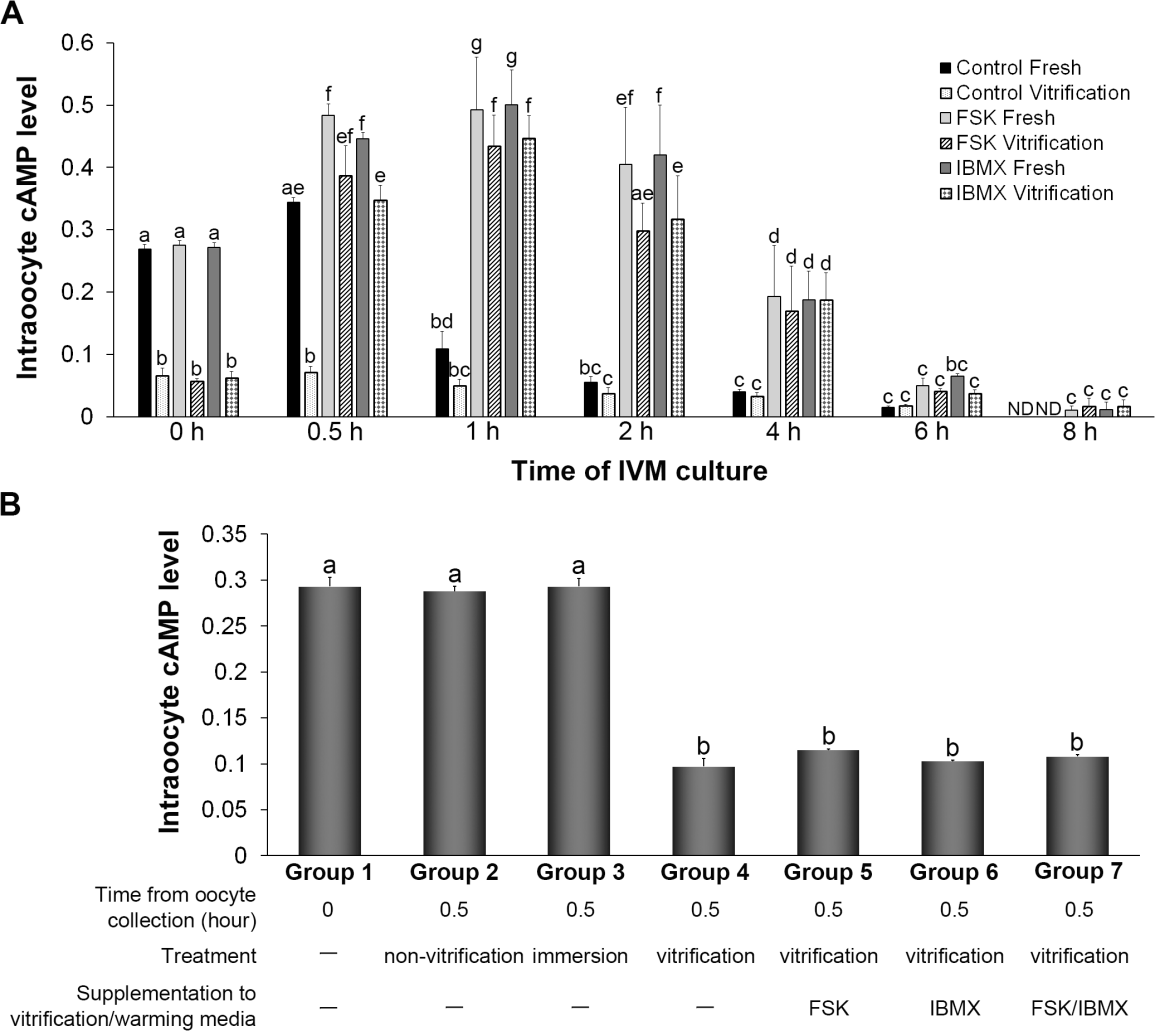
Transient upregulation of cAMP levels in vitrified-warmed germinal vesicle-stage (GV) oocytes treated with forskolin (FSK) or 3-isobutyl-1-methylxanthine (IBMX). *A)* Effect of FSK and IBMX on the level of intraoocyte cAMP during IVM culture. The intraoocyte cAMP level was reduced by vitrification prior to IVM culture, and this low cAMP level could be increased back to normal by supplementation of the IVM medium with FSK or IBMX. *B)* Level of intraoocyte cAMP at the initiation of culture (Group 1) or after culturing for 0.5 h (Group 2), immersion of vitrification/warming solution (Group 3), vitrification and warming (Group 4), vitrification and warming in vitrification/warming solution supplemented with FSK, IBMX or the combination of FSK and IBMX (Group 5–7). The decline in the cAMP level was induced by the vitrification procedure. Non-vitrification, GV oocytes cultured in mTCM199/20%SSS for 0.5 h; immersion, GV oocytes immersed in the vitrification/warming solution; vitrification, GV oocytes vitrified and warmed. Error bars represent standard error of the mean. Bars labeled with different letters show significant differences (*P* < 0.05).

As shown in Fig 2A, a high level of cAMP was observed already in the fresh control group prior to IVM, and the level was significantly decreased 1 h after the initiation of IVM culture. In addition, in the fresh FSK and IBMX groups, the levels of cAMP were significantly elevated 0.5 h after initiation of IVM culture and then started to decrease 2 h after the initiation of IVM culture. The decrease of the cAMP level in the fresh FSK and IBMX groups lagged 4–6 h behind the decrease in the fresh control group. In the vitrified-warmed control group, the cAMP level was already lower than that in the fresh control group and no elevation was observed during IVM. In contrast, in the vitrified-warmed FSK and IBMX groups, cAMP levels were elevated 0.5 h after the initiation of IVM and started to decrease 2 h after the initiation of IVM. These patterns of cAMP alterations were similar to those of the fresh FSK and IBMX groups. These results indicated that vitrification at the GV stage affected the levels of cAMP when the oocytes were warmed, but the levels could be returned to normal with a 4- to 6-h delay by addition of FSK or IBMX to IVM media during IVM.

The cAMP levels of GV oocytes cultured in mTCM-199/20% SSS for 0.5 h (Group 2) and GV oocytes immersed in vitrification and warming solution (Group 3) were similar to those of GV oocytes collected at the initiation of IVM culture (Group 1), but vitrified-warmed GV oocytes (Group 4) showed a significantly lower level of cAMP (Fig 2B). Similarly, a significantly lower cAMP level was observed in GV oocytes that were vitrified and warmed using FSK, IBMX or the combination of FSK and IBMX in the vitrification/warming solution (Group 5–7). These results demonstrated that the decline in cAMP content was associated with the vitrification/warming procedure but was not related to the duration of the procedure or immersion in the vitrification/warming solution. Furthermore, this decline was not restored by the addition of FSK and IBMX to the vitrification/warming solution.

### Initiation of MPF elevation was delayed in vitrified-warmed GV oocytes upon FSK or IBMX treatment

**Fig 3:**
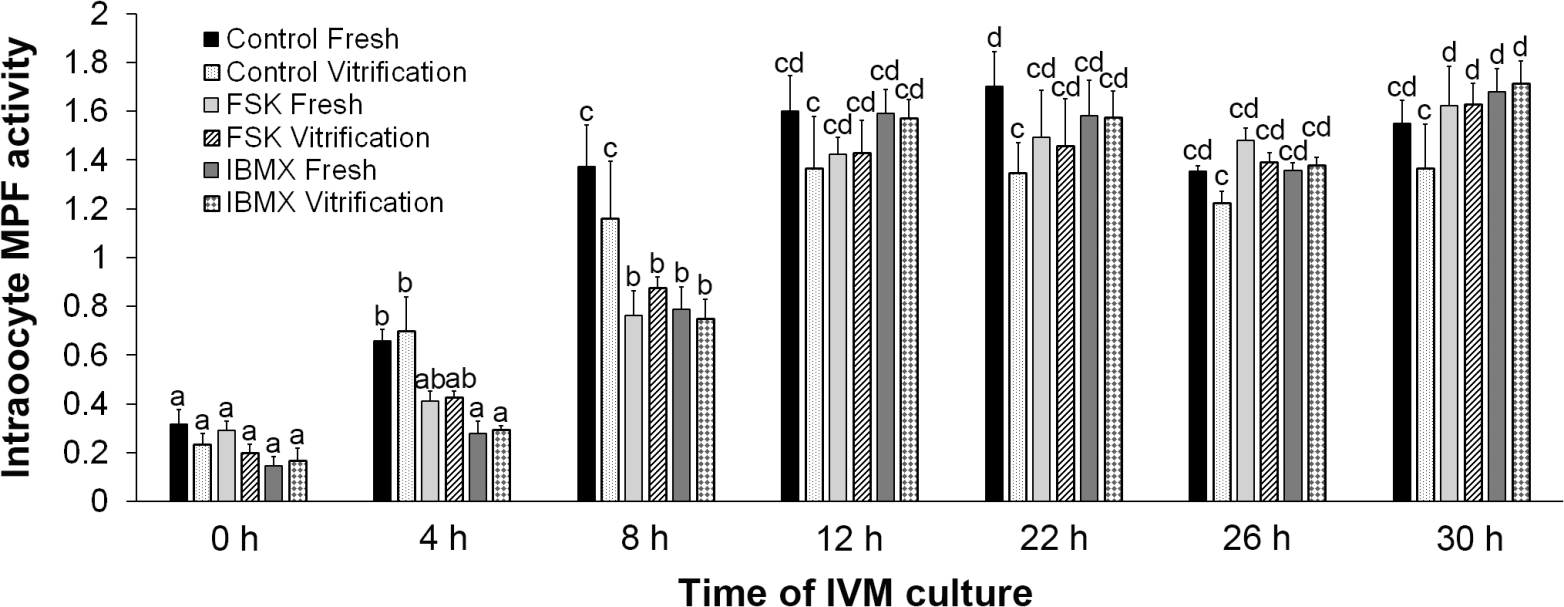
Delayed MPF elevation induced by the treatment of vitrified-warmed GV oocytes with forskolin (FSK) or 3-isobutyl-1-methylxanthine (IBMX). Effect of FSK and IBMX on intraoocyte MPF activity during IVM culture. Low MPF activity was observed in the vitrified-warmed control group at the end of IVM culture, whereas the MPF activity of the vitrified-warmed FSK and IBMX groups was comparable to that of the corresponding fresh groups. Error bars represent standard error of the mean. Bars labeled with different letters show significant differences (*P* < 0.05).

The MPF activity of vitrified-warmed oocytes was measured to determine whether the MPF activation in vitrified-warmed oocytes was delayed by the treatment of FSK or IBMX. The MPF activity of control fresh GV oocytes had already plateaued at 22 h after the initiation of IVM. In contrast, in both fresh and vitrified-warmed FSK and IBMX groups, MPF activity was relatively lower than that of the control fresh group at 22 h and was elevated to a level comparable to that of the control fresh group at 30 h. The MPF activity of vitrified-warmed GV oocytes plateaued at 22 h, but the values were significantly lower than those in other groups (Fig 3). These results demonstrated that recovery of cAMP content by FSK and IBMX was associated with elevation of MPF activity and that this elevation was delayed 8 h.

## Discussion

The present study demonstrates that vitrification of GV oocytes causes a decline in cAMP levels inside oocytes and low MPF activity after IVM culture. Maintenance of the intraoocyte cAMP level during IVM improves poor developmental competence caused by vitrification of oocytes at the GV stage.

cAMP, which acts as an intracellular messenger for gonadotropin stimulation, is synthesized in oocytes and is also supplied to oocytes by cumulus cells via gap junctions [37,38]. During the maturation process, the preovulatory surge of luteinizing hormone decreases cyclic guanosine monophosphate concentrations in granulosa cells and oocytes; this is followed by activation of phosphodiesterase 3A, which causes degradation of cAMP. This process initiates the activation of MPF and the resumption of meiosis. In the present study, high cAMP levels were observed in fresh GV oocytes after retrieval, but these levels declined after vitrification/warming. Recent studies demonstrated that vitrification causes transient membrane damage and cytoplasmic leakage [39], suggesting that vitrification-induced cAMP decline was most likely due to the cytoplasmic leakage through damaged cell membrane. It is well documented that the loss of intra-oocyte cAMP before IVM leads to impairment of developmental competence [13] and the culture of GV oocytes before or during IVM in an environment where meiosis is regulated by a cAMP modulator enhances oocyte maturation and developmental competence [9–14,40–44]. These improvements were achieved either by preventing cAMP degradation using a phosphodiesterase inhibitor, e.g., IBMX, or by increasing cAMP concentrations using an adenylate cyclase activator, e.g., FSK, during IVM [8,9,12–14,45,46]. In the present study, increased cAMP levels were observed in both fresh and vitrified-warmed oocytes 0.5 h after culturing in IBMX- or FSK-supplemented IVM medium. However, the addition of cAMP modulators to vitrification/warming solution didn’t increase the cAMP levels of vitrified-warmed oocytes. These results suggest that the cAMP modulator supplementation of the IVM medium increased cAMP content, but the supplementation of the media during vitrification and warming does not effectively increase cAMP content. Our results also show that a high cAMP level in fresh GV oocytes drastically declines after the initiation of IVM culture. In contrast, cAMP levels of FSK- and IBMX-treated GV oocytes gradually decrease, and this decline lags 4–6 h behind that in fresh GV oocytes. This phenomenon is likely to result a loss of biological activity of FSK and IBMX.

The transient decrease in the cAMP level in oocytes is an obligatory step in the induction of meiotic maturation that is started by GVBD. In GV oocytes treated with IBMX or FSK, the onset of the decline in the cAMP level is delayed compared to that in untreated oocytes. Thus, we hypothesized a consequent delay of GVBD and elevation of MPF in FSK or IBMX treated GV oocytes. As expected, the delayed GVBD is observed in GV oocytes treated with IBMX or FSK similar with the results reported previously [12,41,47], but the incidence of GVBD is similar to that of the untreated oocytes.

MPF performs a dominant function in the process of meiotic maturation. The MPF activity is suppressed to a low level in immature oocytes, and GVBD is induced by the activation of MPF [48,49]. Fusion or microinjection experiments between meiotically incompetent and competent oocytes demonstrated that active MPF from a competent oocyte can induce condensation of chromatin and disintegration of the nuclear membrane in an immature oocyte [50]. Therefore, the ability of the oocyte to resume meiosis is associated with the MPF activity. Our results show that there is no difference in the maturation rate but that MII oocytes derived from vitrified-warmed oocytes have lower MPF activity than fresh oocytes. This effect suggests that the deficiency of cyclin B synthesis or early destruction may occur at the MII stage, or that defective phosphorylation of threonine or serine residues can affect formation of the active form of the MPF complex. A decrease in MPF levels has also been reported in oocytes that have low developmental competence, such as aged oocytes and oocytes cultured under suboptimal conditions [51]. These data suggest that deficient MPF activity is one of the causes of the reduced developmental competence of vitrified-warmed oocytes at the GV stage. Furthermore, the addition of FSK and IBMX to IVM medium increased MPF activity in vitrified-warmed GV oocytes to a level similar to that of the fresh GV oocytes (according to our present results), which may result in transient inhibition of the MPF activation by elevating the cAMP level followed by an increase in MPF activity caused by decline in cAMP concentration. Our results suggest that restoration of the cAMP level by FSK and IBMX stimulates the MPF activity and improves the impaired developmental competence of vitrified-warmed oocytes at the GV stage.

Techniques for cryopreservation of oocytes offer women with cancer the chance to preserve their fertility. The cryopreservation of mature MII oocytes has been the mainstream regimen in clinical practice until now. One of the reasons why MII oocytes have been favored is the tendency for decreased developmental competence of GV oocytes. The present study shows that vitrification induces impairment of developmental competence but that this can be recovered by supplementation of the IVM medium with FSK or IBMX. This improvement may be associated with an increase in MPF activity induced by the restored cAMP levels. Therefore, these findings may provide clues to the improvement of GV oocyte cryopreservation regimens in clinical practice.
